# The subjective experience of collaboration in interprofessional tutor teams: A qualitative study

**DOI:** 10.3205/zma001024

**Published:** 2016-04-29

**Authors:** Tobias Weber, Henriette Hoffmann

**Affiliations:** 1 Technische Universität Dresden, Medizinische Fakultät Carl Gustav Carus, Medizinisches Interprofessionelles Trainingszentrum im Referat Lehre, Dresden, Germany

**Keywords:** Interprofessional Relations, medical education, Peer Tutoring, Teaching and Learning, Social Identification

## Abstract

**Aim:** The Center for Interprofessional Training in Medicine at the Faculty of Medicine Carl Gustav Carus at the Technische Universität Dresden, Germany, has offered courses covering interprofessional material since the winter semester 2014/15. The unusual feature of these courses is that they are co-taught by peer tutors from medicine and nursing. This study investigates the subjective experiences of these tutors during the collaborative preparation and teaching of these tutorials with the aim of identifying the effects of equal participation in the perceptions and assessments of the other professional group.

**Method: **Semi-structured, guideline-based interviews were held with six randomly selected tutors. The interviews were analyzed using structuring content analysis.

**Results: **The results show that collaborative work led to reflection, mostly by the university student tutors, on the attitudes held. However, the co-tutors from each professional group were perceived to different degrees as being representative of those in their profession. Asked to master a shared assignment in a non-clinical context, the members of the different professional groups met on equal footing, even if the medical students had already gathered more teaching experience and thus mostly assumed a mentoring role over the course of working on and realizing the teaching units. The nursing tutors were primarily focused on their role as tutor. Both professional groups emphasized that prior to the collaboration they had an insufficient or no idea about the theoretical knowledge or practical skills of the other professional group. Overall, the project was rated as beneficial, and interprofessional education was endorsed.

**Conclusion: **In the discussion, recommendations based on the insights are made for joint tutor training of both professional groups. According to these recommendations, harmonizing the teaching abilities of all tutors is essential to ensure equality during cooperation. Ideally, training programs should be attended together by medical and nursing students to emphasize their shared identity as “tutor”.

## Zusammenfassung

**Zielsetzung: **Das Medizinische Interprofessionelle Trainingszentrum der Medizinischen Fakultät Carl Gustav Carus an der Technischen Universität Dresden bietet seit dem Wintersemester 2014/2015 Lehrveranstaltungen mit interprofessionellen Inhalten an. Die Besonderheit dieser Lehreinheiten besteht darin, dass sowohl studentische TutorInnen der Medizin als auch SchülertutorInnen der Gesundheits- und Krankenpflege gemeinsam die Lehreinheiten betreuen. Die Studie untersucht das subjektive Erleben der TutorInnen während der gemeinsamen Ausarbeitung und Durchführung dieser Lehreinheiten mit dem Ziel, die Effekte der gleichberechtigten Zusammenarbeit auf die Wahrnehmung und Einschätzung der jeweils anderen Berufsgruppe herauszuarbeiten.

**Methode: **Es wurden teilstrukturierte Leitfadeninterviews mit sechs zufällig ausgewählten TutorInnen durchgeführt. Diese werden mittels inhaltlich-strukturierender Inhaltsanalyse ausgewertet.

**Ergebnisse: **Die Ergebnisse zeigen, dass das gemeinsame Arbeiten vor allem bei den studentischen TutorInnen zu einer Reflexion bestehender Einstellungen geführt hat, jedoch wurden die jeweiligen Co-TutorInnen bei beiden Berufsgruppen in unterschiedlichem Grad als Repräsentanten ihrer Profession wahrgenommen. Durch die Bewältigung einer gemeinsamen Aufgabe in einem nicht-klinischen Kontext begegneten sich die Angehörigen der verschiedenen Berufsgruppen auf Augenhöhe, wenngleich die Medizinstudierenden bereits mehr didaktische Erfahrung aufwiesen und somit im Zuge der Erarbeitung und der Umsetzung der Lehreinheiten meist eine Mentoren-Rolle übernahmen. Die SchülertutorInnen waren vorwiegend auf ihre Rolle als TutorIn konzentriert. Hervorgehoben wurde von beiden Berufsgruppen, dass sie vor der Zusammenarbeit mangelnde oder keine Vorstellungen bezüglich des theoretischen Wissens und der praktischen Fertigkeiten der jeweils anderen Berufsgruppe besaßen. Das Projekt insgesamt wurde als gewinnbringend eingeschätzt und der Ansatz der interprofessionellen Lehre befürwortet.

**Schlussfolgerung: **In der Diskussion werden aus den Erkenntnissen gewonnene Empfehlungen für die gemeinsame Tutorenausbildung beider Berufsgruppen gegeben. Demnach ist eine Angleichung der didaktischen Kompetenz bei allen TutorInnen unerlässlich, wodurch ein gleichberechtigtes Zusammenarbeiten gewährleistet werden soll. Die Schulungen sind idealerweise gemeinsam zu durchlaufen und das verbindende Element des „TutorIn-Seins“ zu betonen.

## 1. Introduction

Successful interprofessional collaboration (IPC) [1] is considered to be a quality-increasing factor in patient care and job satisfaction among health care workers; it is viewed as a key element for meeting the ever growing demands and challenges facing the health care system [2], [3]. Important aspects of IPC include a shared understanding of the division of labor, roles and work processes, as well as knowledge of the capabilities and skills of the other health care professions [4]. According to a thesis formulated by Sieger et al. [5], the ability to engage in IPC is acquired through interprofessional learning (IPL). The German Advisory Council on the Assessment of Developments in the Healthcare System subscribes to this and recommends “gear[ing] training in all health care professions to action on a common object” [6]. The basic conditions for achieving this hardly exist at present since the socialization of the medical, nursing, therapeutic and diagnostic professions during education and training occur separately for the most part [[7], p. 12]. IPL may be talked about by all, but the few teaching projects addressing this issue demonstrate great differences in quality and quantity despite any shared intentions. Examples of this variance are the projects on teaching interprofessional ethics and teamwork as an elective subject [8], [9]. Alongside projects that cover individual, topic-specific courses, the academic inclusion of different nursing professions is being promoted, as a result of the “academization of nursing” [10], and culminating, among other things, in interprofessional model study programs. One instance of this is the bachelor degree program in Interprofessional Health Care [11]. Undergraduate nursing education continues to remain within the sphere of the post-secondary vocational schools, something that impedes the establishment of interprofessional courses in terms of their organization and structure despite the existence of a model clause in the regulations governing the profession of nursing and geriatric care allowing the conduction of model projects outside of these vocational schools as long as the educational objective stipulated by law is not endangered [10]. However, if the only opportunity for contact among students of the various professions remains in these isolated instances, the academic world of the university will remain unknown and strange to vocational students, and any basis for interprofessional collaboration, for both peer tutors and tutees, must be initially negotiated in direct interaction.

Currently, there are no formally defined skills or responsibilities for those teaching interprofessional courses and a lack of standardized (post-licensure) educational programs. However, it is asserted that it is imperative to develop and implement interprofessional learning strategies in interprofessional teams [[7], p.15]. According to Gardner et al. [12], the following constitutes opposition to the establishment and integration of interprofessional education: jealous guarding of professional terrains, lack of trust, lack of knowledge about group dynamics, differing values, professional ethics, resistance to change, and lack of space in the curriculum. A position paper by the GMA Committee on Interprofessional Education in the Health Professions states that to date “no substantiated knowledge about the effects of interprofessional learning situations exists yet for students of the various health professions” [[7], p.7]. 

The Center for Interprofessional Training in Medicine (MITZ) at the Faculty of Medicine Carl Gustav Carus at the Technische Universität Dresden, Germany, has since the winter semester 2014/15 offered special tutorials covering interprofessional content. In addition to the focus on interprofessional aspects of medical and communication-related content, the special feature of the course is the composition of its tutor teams which are each made up of one medical student and one student from nursing or pediatric nursing. The courses follow the peer-teaching approach.

For the projects cited above and in the courses offered at MITZ, evaluations by the student participants were usually drawn upon to assess the relevance and success of the interprofessional units. The emphasis of this study, however, is on the cooperation of the interprofessional tutor teams. Our aim is to document and analyze the process of becoming acquainted and working together in the tutor groups over the course of preparing for the tutorials. The issue here involves the subjective experience of the tutors: how do the medical and nursing students perceive the cooperative work and teaching the courses, and what are their reflections on their existing attitudes about the other professional group and their own. This should provide information on the particulars that must be taken into account regarding the cooperation of mixed tutor teams and the factors that foster cooperation.

To accomplish this aim, structured interviews with the tutors were conducted after the course ended to gain insights on the process of becoming acquainted, reflection of possible prejudices at the personal level, how decisions were made by the team, and teaching. Since the first-time offering of the interprofessional courses during the winter semester 2014/15 involved a pilot project, the data collected from it concerning team processes can only be viewed as exploratory in nature. In our discussion, various aspects will be referred to that make generalizations difficult and which should be taken into consideration by further studies. Based on the knowledge gained from this study, recommendations are made in the final section for future strategies for offering courses taught by interprofessional tutor teams.

## 2. Interprofessional courses at MITZ

After two block courses in 2011 and 2012, which required substantial human resources, a new concept was developed in 2014 with the goal of reintroducing interprofessional education in a manner that conserves resources. The lack of space in the curriculum was addressed by MITZ by integrating interprofessional education into the required medical curriculum on separate dates covering an additional topic: interprofessional learning. Students were able to choose if they wished to complete the normal training in the ninth semester of study or the enhanced course units. MITZ decided to prioritize imparting knowledge about spheres of responsibility and capabilities of one’s own and the other professions and raising awareness about interprofessionalism, instead of the skills for successful interprofessional collaboration. The ninth-semester training showed itself to be a very promising application of interprofessional subject matter since procedures in patient care that are carried out jointly are taught later. During the ninth semester, medical students cover units *on Port placement, Informing about surgical operations, Inserting a stomach tube, Motivational Interviewing, Respiratory management, *and* Seldinger technique for central line placement* *(CVC)*. The interprofessional training itself took place in small groups with two medical students and two vocational students and was taught by the interprofessional tutor team. In total, 42 medical students and 33 vocational students attended the interprofessional tutorials. An equal balance between the two professions within the small groups was not always possible due to the difference in the overall numbers of attendees.

**Figure 1:**
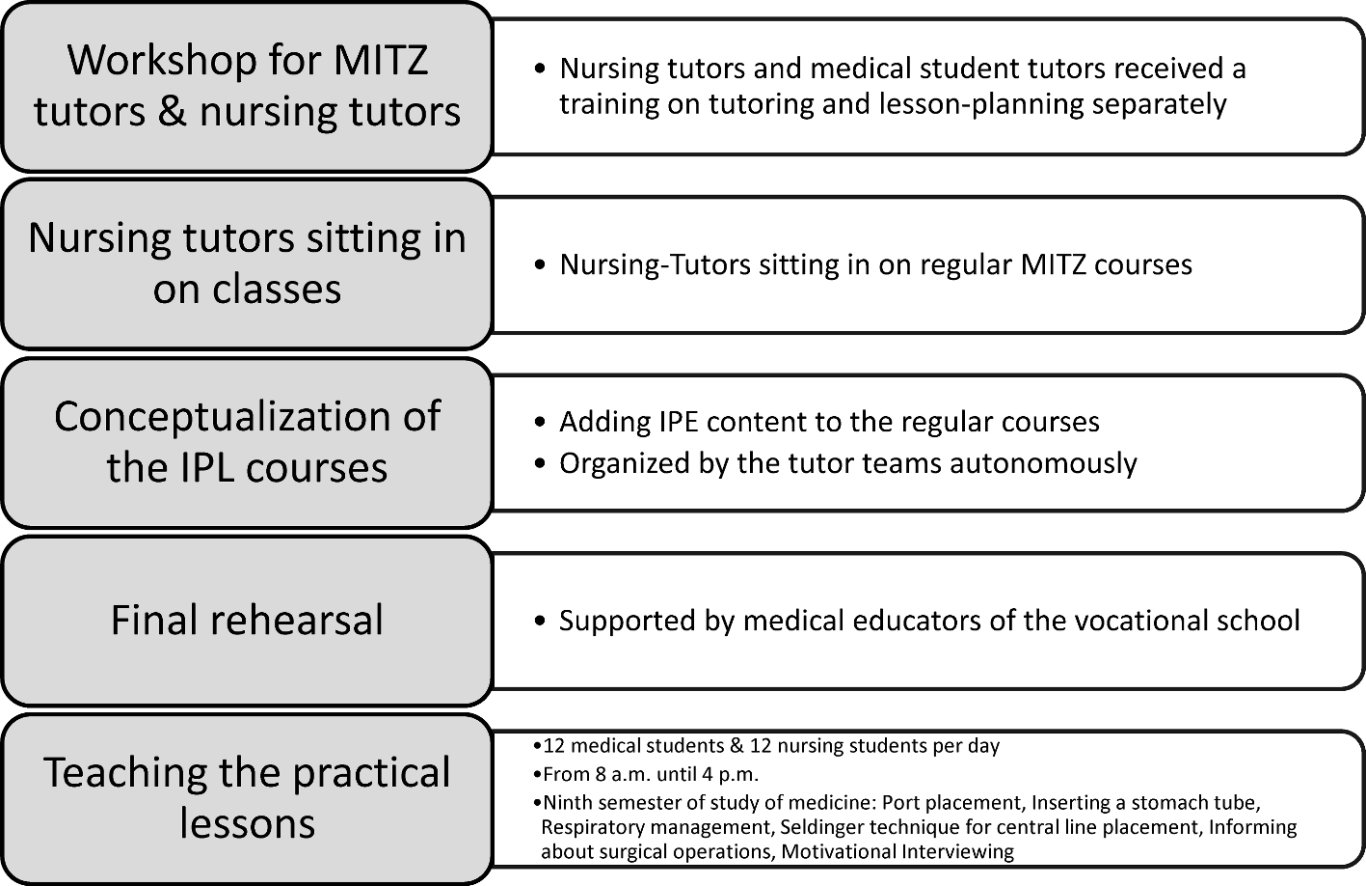
Sequence of tutor-training, conceptualization, and teaching of the IPL-lessons at MITZ

As part of preparation, vocational students who demonstrated an interest in and aptitude for teaching were selected from the Carus Academy at the University Hospital Carl Gustav Carus in Dresden, Germany. These student tutors received training at MITZ on tutoring and lesson planning. Since the medical student tutors had already undergone this training, this step was separate for the two professional groups. The initial meeting for all tutors took place at the start of the conceptual phase for the individual tutorials. The tutor training and the design of the stations up through teaching the lesson is presented in figure 1 (Fig. 1).

## 3. Method

Due to the explorative character of the analysis of the personal and social processes while designing and teaching the lessons, semi-structured interviews were held as part of this study. Questions asked by the interviewer aimed toward eliciting deeper insights and personal meaning as a pre-requisite for appropriate interpretation [[13], p. 425]. Open-ended or unstructured interviews appeared inefficient in light of the specificity of the experiences. The structure of the interviews was created by the researchers involved and encompassed three major topics:

1. Evaluation of the overall project and its principle idea
2. Understanding one’s own role and interacting with the other tutors with respect to professional group
3. Evaluation of one’s own role and work as tutor

The first part of the interview asked about the interviewee’s understanding of interprofessional learning (IPL) to understand the statements as being an agreement with or a (partial) rejection of the teaching approach. The second topic explicitly addressed the issue under investigation in this study in which the focus is on membership to a particular profession. The final part asked for an assessment by the interviewee from the perspective of a tutor. Since there were no structural differences for the tutors in conceptualizing and teaching their course-unit, the subjective differences of experience are of peculiar interest for this study.

Of the 22 tutors who taught the course, three were selected randomly (computer-generated random numbers) from each professional group to be interviewed individually. A quota of professional groups was necessary to ensure that neither group was over-represented or that differences within the groups were overlooked. An interview was held in advance with one tutor who was not among those randomly selected. Afterward, necessary changes were made to the interview’s structure. Two interviewers were responsible for conducting the interviews who did not participate in the design or conduction of the tutor training program or any supervision. This involved a male and female interviewer. The aim was to avoid interviewer effects arising from shared experiences or a specific gender constellation.

The questions were largely open-ended giving the opportunity to analyze the content and the structure of the responses. Some questions were asked in a closed manner at first, an example of this being, “Do you view your co-tutor as a typical representative of their professional group?” If a short reply was given, the interviewer had been instructed to ask follow-up questions and encourage open-ended responses. 

Analysis was carried out by first compiling the responses to the questions posed by the interviewer into an overview. This enabled not only a reconstruction of an individual interview, but also a comparison of the different responses to the same question. Recurring topics and responses, as well as differences, could be clearly identified (structuring content analysis). The statements were then transcribed for interpretation and to discover underlying connections.

The quotes cited from the interviews were transcribed simply, meaning that slang and regional dialects have been smoothed over. Furthermore, prosodic or nonverbal features were not recorded. This loss of information was accepted for the sake of concentrating on content and argumentative coherency and ultimately better legibility of the statements [14]]. The quotes are anonymous. Characteristics of the interviewees which could be important in categorizing the statements have been listed in table 1 (Tab. 1). The interviews varied in terms of length ranging from 21 to 37 minutes (M=30.5 minutes).

## 4. Results

In the following section we present the interview results in the order of the major topics. The quotes from the interviews are included in part as illustrations of the summarized content, but in some cases particularly interesting assertions are considered and interpreted.

### 4.1. .Evaluation of the project and the underlying concept

The first questions referred to the project in general and the underlying idea of interprofessional cooperation. The university student tutors who were interviewed understood the basic concept of interprofessional tutorials to be different professions learning together at an early stage of their education to promote future collaboration in day-to-day practice. It was emphasized that the situation here allowed interaction without the constraints or demands of professional life (interview 2). One interview addressed the idea of taking a different point of view and becoming familiar with the perspectives of other professions (interview 6). One interviewee referred to the great dependency of the professional groups on each other in routine hospital work (interview 1). In the statements made by some tutors the idea was expressed that conflicts exist not just within professional groups, but also between them, and that these all compromise productivity (interview 1, interview 2).

There are many power struggles, first between the nurses and orderlies and then also between the physicians and nursing staff, and it is very, very important for everyone to get along well because it is very helpful (interview 2).

This tutor explicitly said that there are power struggles not only within the nursing profession, but that interprofessional conflicts also exist. She stresses the importance of understanding each other. However, it is unclear if she (only) means the cooperation or also aspects related to group dynamics at a personal level. The relative clause, “because it is very helpful,” can be interpreted in both ways as referring to effectively achieving a goal or the social resources pertinent to daily work on the hospital ward. All of these aspects, meaning the power struggles, the interdependence, and advantages of effective cooperation are explicitly highlighted in the literature on interprofessional collaboration (IPC) [4], [15].

The vocational student tutors seemed to focus more on the imminent task of tutoring (interview 3). The meeting of the vocational and university students was rated positively by the vocational students (interview 3); the idea of interprofessional collaboration or any associated conflicts were not mentioned by them. Only one vocational student referred to her own experiences interacting with other professional groups in a ward setting (interview 5). This is remarkable since, as a result of having more experience in daily ward activities, the vocational students should be more aware of interprofessional collaboration as a topic than medical students, who acquire their theoretical knowledge in a closed group setting and gather practical experience during very brief time periods (e.g. three month-long nursing internship). Above all, the vocational students cited equality and mutual respect as a special aspect of the teaching situation (interview 4), something that not only refers to the actual teaching situation, but also considers the basic idea of IPC in terms of dividing tasks according to different responsibilities [16], [17].

The basic concept of IPL was accepted to a great extent by all the interviewees, and the focus of the project was endorsed. Individual aspects of the organization were criticized by the medical student tutors, primarily the brief preparatory time, scheduling the sessions in the middle of studying for exams, and the partial lack of communication among the tutors and those responsible for subject areas. The vocational student tutors complained about the lack of information about specific procedures and criticized preparation. Tutor training was seen positively and evaluated as sufficient. None of the problems mentioned were viewed as endangering the project.

#### 4.2..Understanding roles and interaction with the other tutors based on professional group

The university student tutors were asked to list general traits which they spontaneously associated with members of the nursing profession. In their responses, students mostly referred to their own practical experiences (clerkships), indicating that their attitudes do not involve an elaborately abstracted concept of the other profession, but rather an assessment that is constructed and supported using personal experience. These experiences prove to be very heterogeneous:

Some were really good experiences in which I was accepted even as a student since there was a certain level of respect and there were people with whom it was possible to work with really well. And there was a really great atmosphere. On the other hand, I often noticed that others just had to remind me that I was still just a student and give me a good dressing down and really let me flounder without any help. In those situations, you have to defend yourself, and I found that extremely unpleasant. At the beginning there was a barrier, and I felt from the very start that I didn’t want to have anything to do with these others (interview 1).

This student refers, in part, to contrary experiences. To evaluate these situations, the aspects of integration, esteem, and cooperation/work atmosphere seem to be of great importance. If one of these aspects is felt to be lacking, the interviewee defiantly reports that he didn’t want to have anything to do with them. The reflection on these experiences is very differentiated. As soon as generalizations were made, they were relativized as follows:

They were often of an older generation, although I have had very positive experiences with older nurses. It is always difficult, generalizations can’t be made (interview 1).

With this assertion, the student gives the impression that certain stereotypes implicitly exist (older nurses who make things difficult for inexperienced medical students to maintain their status), which are then revised in practice and lead to the insight (“generalizations can’t be made”).

In general, nurses were seen as being very nice and friendly. This applies to most statements primarily in regard to interaction with patients. Negative assessments were in turn differentiated and not general:

There are friendly nurses, yes I would call them friendly, but there are also ones who are beastly shrews (interview 2).

As in interview 1, this statement could implicitly indicate a stereotype; however, the stereotype in this case remains less concrete.

Alongside attributing character traits, aspects relevant to decision-making and taking action were mentioned. One interviewee shared that some nurses gave him the impression they only do what they have to and complain too much (interview 1). In comparison with this, one female tutor described the nurses as active and taking initiative (interview 6). It is nothworthy that, due to the different experiences, the general appraisals are to some extent oppositional. Other information reported by the university student tutors about working with their nursing co-tutors suggests that their assessments of the professional group were heavily affected by their experiences cooperating on the IPL project. Moreover, phenomena arising from group dynamics were mentioned:

But you shouldn’t turn away from them too much; otherwise you will have problems (interview 1).

Based on the prior statements concerning experience, this one is very general and almost formulaic: “one should/should not do this, otherwise there will be problems.”

This demonstrates an interesting aspect of daily work on a ward as it is expected or experienced to be. Nursing is perceived by the medical students as positive, mainly in the area of personal interaction, but also in a differentiated manner interpersonally. Working on a hospital ward with all the attendant phenomena previously mentioned, such as hierarchy, power struggles and group dynamics, is viewed as a closed system; everyone involved is part of the group and acts accordingly.

Questions were not directly asked about assessments of one’s own professional group, but this was addressed in different contexts during the interviews. One interviewee spoke of the theoretical superiority and practical inferiority of physicians in comparison with nurses:

Everyone knows that doctors don’t like to do practical stuff; they think they can do it all anyway (interview 6).

In this vein, the university student also touched on the over-confidence of physicians saying, they can do everything. The introductory phrase, “everyone knows,” points to an accepted and generalized perception.

Among the nurses, it is emphasized that physicians have more theoretical knowledge and carry more responsibility (interview 4). This characterization shows a mix of institutionally defined traits associated with the role (responsibility) and personal attributes such as medical expertise (deriving from the fact of university study) or self-confidence (interview 4) and readiness to help (interview 3). Differences within the professional group of doctors are not specified by the nursing students.

It is conspicuous that in the responses to the question about the traits of the other professional group, the university student tutors mentioned both structural characteristics – nurses have more proximity to patients based on their work and this leads, above all, to advantages regarding information. The nurses are the closest contact person and are, in part, in a better position to assess the overall condition of the patient than the attending physician [interview 6] – and personal characteristics, such as beastly or friendly. The question as to whether these assessments involved attributing a personal trait or behavior that was governed by the situation or organizational structure should also be asked. In connection with this, the effects of this characterization on the views of the co-tutor must be examined. In order to categorize these properly, the tutors were asked to what extent they found their co-tutors to be representative of the other professional group and were prompted to describe their interactive experiences.

The first two I worked with, they were not really like that, I would say. They were quite clever, I’d say, and did quite a lot by themselves without seeming to be dominant (interview 1).

The student bases his assessment on a specific feature (self-initiative). Since he does not view his co-tutor as representative, he does not attribute this feature to nursing in general, and if he does, then as a form of dominance. This corresponds with an earlier statement made by this tutor in which nurses only do what they have to.

Ty*pically, yes, very open, very people-friendly, very active. Also not particularly inhibited like many doctors are – shy and the like – that’s how I imagine it (interview 6).*

As the quote immediately above demonstrates, another medical student tutor expresses her perceptions in a way that distinguishes nurses from doctors.

It was necessary to critically examine to what degree the professional group or the group of participants undergoing training was meant, since the term “shy doctors” could potentially refer to the medical students and not to full-fledged physicians.

Tutor: I see them (nursing students) as students, but since we are working on another project together they don’t have this function on the ward in my head.

Interviewer: Meaning you saw them more as tutors than nursing students; you worked with them on equal footing?

Tutor: Precisely (interview 2).

The interviewee did not affirm the question about the representativity of the nursing student tutors and referred with emphasis to the unusual context of the cooperative work. In the diverse ways interviewees responded to the questions it can be seen that the given context and the joint tasks have an influence not only on the assessments of the co-tutors, but also seem to affect the extent to which they were considered to be representative for their particular professional group. The heterogeneity of this pattern of justification can be interpreted as a consequence of the novelty of the situation.

Nursing students responded to this question in a variety of ways with a pattern emerging in the answers. The medical student tutors are seen as representative in terms of their medical expertise, but this is reversed if these tutors were being described on a personal or social level:

She (the medical student tutor) didn’t give any sign that I was “just” the nursing student, instead we got along really well and liked each other from the start and were on the same wave length, something I do not always experience now in my daily work (interview 4).

This general assessment of physicians by the nurses did not contain any negative traits attributed to the physicians personally; this is implicitly revised in the quote immediately above (derived from the statement is the tacit assertion that there are doctors who make it clear to me that I am only the nursing student). This missing attribution of negative traits can be viewed in two ways: that the hierarchy, such as it is, is accepted as an institutional reality or that the propagation of hierarchical structures is only implicitly attributed to physicians and that they are not consciously responsible for it. Both interpretations are plausible based on the mix of personal and institutional traits described in this paper. The topic of hierarchy and esteem appear to play a major role for nursing staff since they feel directly affected by this and the special feature of the project is increasingly seen in the mutually respectful work of the nursing and medical student tutors. This corresponds with findings according to which nurses indicated that job dissatisfaction primarily comes from a lack of appreciation [18].

The collaborative work of the tutor groups was valued throughout, and all interviewees felt their co-tutor had been supportive. Medical students’ perception of support from the nursing students concentrated on content-related aspects. It was particularly pointed out that the two tutors mutually complemented each other. In some cases, the productive cooperation was described as the culmination of a learning and adjustment process (interview 2, interview 5). In these cases it appears that in the beginning there was an imbalance between the medical student and the nursing student which can be explained as a result of the different educational experiences and the, in part, unspecific content and objectives of the separate teaching units on the part of the nursing school. This was expressed through a certain reservation and nervousness seen in some nursing students (interview 2).

The medical students showed the necessary understanding for this issue and put the nursing students’ fears to rest (interview 5), whereby the nursing students perceived this as a form of support. Elaborate and repeated feedback during preparation and teaching was viewed as an essential aspect of this support (interview 4).

No explicit conflicts were mentioned. Minor differences referred exclusively to organizational aspects (communication, scheduling meetings). During this period of adjustment, improvements relating to content or teaching were suggested mostly by the medical student tutors:

I mentioned a few things, particularly to the second group, that they should come out of their shells a bit more, be a little more awake and alert (interview 2).

The university student described these comments to the co-tutor as recommendations that did not refer to content or knowledge-related aspects. It is striking that it refers in part to certain behavior (coming out of one’s shell), and in part to a basic attitude (being alert and involved).

Exchange among the tutors mainly took place within the professional groups or within the tutor teams of a specific tutorial. If tutors did not belong to the same professional group or teach the same lesson, establishing contact was difficult.

In terms of the IPL sessions I mostly dealt with my co-tutor and otherwise with the medical students, since I knew them better, and the co-tutors, the others I mean, sat there rather inert and pouty they looked and not very happy sitting there alone (interview 2).

That the most frequent contact was with the co-tutor is due to the structure of the tutorial that called for constant cooperation and proximity while teaching the six sessions. Contact with the medical students is explained by the tutor as being the result of familiarity. However, the interviewee’s description of the impression the other co-tutors made on her is remarkable. Even if it is not explicitly stated, it appears that the description of “inert and pouty” and “unhappy” created a feeling of distance and she did not perceive any need to establish contact. Alongside one’s own professional group (*ingroup*)^1^ there is now a second ingroup comprised of the two co-tutors of a particular tutorial. This binding aspect facilitates the establishment of contact; the general identity of “tutor” appears insufficient for this. According to the minimal group paradigm this could, however, be sufficient if this identity emerges as a very strong criterion for differentiation [19]. For this, a high level of identification with the role of tutor must be present.

I heard that some of the nursing student tutors only became tutors so that they could somehow gain in status ... and that they didn’t really have any actual desire to tutor and that that shouldn’t be the motivation ... For my co-tutor this wasn’t the case; there really was a desire to do it (interview 2).

This statement appears more understandable in this context. Especially the formulation “for my co-tutor this wasn’t the case” refers to a differentiation between the co-tutor and the other co-tutors. This latter group is differentiated in a nonspecific manner (“some”) and negative reasons for participating are attributed to them by an unknown source (“I heard”). The difference between one’s own groups and other groups is also seen in reference to the other tutorial participants, the tutees:

Because I didn’t know these people (the nursing student tutees) at all. I mean, I am familiar with my medical students, I know a bit more about how to act with them. But I don’t want to step on their toes either (interview 1).

Again, the possessive pronoun “my” is used as an expression of familiarity and social belonging.

In the literature addressing IPC knowledge about the capabilities of the other professional groups is considered a core competency for interprofessional collaboration [20]. The tutors became aware of the disadvantage of knowing too little about the expertise and skills of the other professional group:

I* never could quite imagine what the nursing tutees know, what do they already know how to do, can they do more, can they do less; at the start they didn’t speak out much making it hard to figure them out and include them (interview 1).*

This student refers to the complete lack of knowledge about the capabilities of the other professional group. His statement, “I never could quite imagine,” indicates the missing basis for evaluation which most likely has its roots in the separate educational tracks. If poor communication is a topic at the very beginning, then this indicates a search for common starting point where the medical student tutor attempts to meet the participating nursing students, a place from which they can be included and everyone can collectively move forward. During the interviews it was pointed out that the nursing students were more advanced in practical skills. What was surprising in some situations was the equal amount of theoretical knowledge:

I thought it was really good that we were pretty much at the same level, theoretically. That surprised me a little.... And there were times when the nursing students simply knew more, more about interconnections and contexts (interview 3).

This generally contradicts the attitude toward the professional group of physicians that often emphasized the superiority of medical expertise. Upsetting this presumption can lead to medical students not being seen as fully representative of their professional group, yet at this stage of their education and this can, in turn, facilitate interprofessional learning.

#### 4.3..Assessment of one’s own role and work as tutor

The examples above clearly indicate that those interviewed saw themselves more as tutors and only partially as members of their respective professional group. An attempt was made at the beginning of the preparatory phase and teacher training to communicate the special demands placed on tutors in the context of the IPL project. Along with the theoretical aspects, the underlying concepts of joint learning and cooperation need to be exemplified in the behavior of the tutors as teachers. For this reason, the interviewees were requested to list with hindsight the characteristics of a good tutor in the context of an IPL tutorial and to evaluate themselves in this respect.

The medical students considered an interest in cooperating and a willingness to work on an equal footing with the others to be the basic requirement for working with tutors from other professional groups (interview 2), as well as the ability to put one’s self into both roles:

What you then notice is that there is a wall between the two parties so that you then try to make it clear to the other participants that they now have to do something together, that they are permitted to express themselves.... That was a bit more of a challenge (interview 1).

This university student describes the challenge as a wall separating the professional groups. The tutors are to provide support in overcoming this barrier. The interviewee speaks of two different aspects. This involves cooperation (doing something together) and communication, or expression/self-assertion (being permitted to express oneself).

In addition, willingness to delegate tasks and confidence in the capabilities and skills of the other person are named (interview 2). Interaction should be respectful, supportive and polite, for “it is possible to learn from each other” (interview 6). In their responses, the nursing students concentrated on the demands of being a tutor in general in terms of expertise, self-confidence and ability to motivate. Special aspects connected with IPL were not mentioned. What must be taken into account is that the role of tutor was already familiar to the medical students; however, the nursing students found themselves in a completely new situation and had to adjust accordingly, explaining their focus on basic teaching requirements. Moreover, the nursing student tutors had no comparison with a monoprofessional tutorial and were unable to derive insights specifically on IPL from their experience.

I think the biggest hurdle was making what this was about clear to the nursing students because they were unfamiliar with what a ‘station’ is and that they needed to speak up and that, above all, the schedule is difficult to manage (interview 1).

The student here is speaking about the task of informing the nursing student tutors about station-based teaching and of helping them develop teaching skills. Even if this student expresses it generally as making it clear to someone, it does point out that he really means, at least in the beginning, the more experienced tutors assumed mentor roles for the nursing students.

The medical students evaluated the success of their efforts during the tutorial to increase the self-confidence of the nursing students positively (interview 1), and to point out to the medical student tutees that “you need such people in life, in professional life” (interview 1) and it “shows that cooperation is fun” (interview 2).

#### 4.4..Conclusions drawn by the tutors

The interviewees were asked to summarize their experience and make conclusions about their future practices based on their insights on it. The medical students saw the IPL content and cooperation with the other tutors as an enriching experience and evaluated the modified tutorials as being better than the original monoprofessional teaching-units.

Looking to the future, the tutors stressed the confidence they gained from interacting with the other professional group. It is important to seek more contact with each other and to create a positive work atmosphere (interview 1). Shared concepts were emphasized:

There aren’t such big differences between university study and vocational education ... there are certain similarities, in terms of people, educational level and practice. I mean doctors know more about theory, but in terms of practical skills nursing students have already done more (interview 1).

The first part of this statement is surprising because currently the two educational programs still differ very strongly. This assertion seems somewhat confused: at the beginning similarities are mentioned between the two educational programs, but then this is expanded to include between the people and finally in terms of knowledge. The second sentence is contrasted by a statement made in interview 3 that “the nursing students simply knew more, more about interconnections and contexts.” It appears that the views reflect a mix between those studying a profession and those actually practicing a profession.

Communication is viewed even more strongly as a critical tool and precise communication as a pre-requisite for collaborative work (interview 2). By becoming acquainted and interacting with the other tutor, both professional groups come to the conclusion that communication with each other is possible and desired by both sides. Knowing about the capabilities and skills of others results in openness and willingness to learn on the part of the physician and increases mutual respect (interview 6). Another effect is awareness of one’s own attitudes and scrutiny of any prejudice against the other professional group: 

I noticed that there are an incredible number of prejudices on both sides, even me, and that there is a lot of dynamic that is already in people and that perpetuates itself somehow through experience (interview 2).

This tutor is unclear in her comment about whether or not she is referring to previous experiences or specific impressions gained from the tutorials.

## 5. Discussion

The use of mixed tutor teams in an interprofessional tutorial was meant to demonstrate the significance of IPC for both professional groups and sets the foundation for professional collaboration between the tutors. Important aspects of IPC, as described in the first section, could indeed be realized. Focus was primarily on the shared tasks, negotiating roles, knowledge about the capabilities and skills of the other, and securing the results while the tutorials were being conducted. The results clearly show that contact and cooperation between the university and vocational student tutors are influenced by both general phenomena of group dynamics (e.g. ingroup/outgroup distinctions) and specific aspects of interprofessional collaboration in medicine (e.g. occupational socialization, practical or teaching experience). The medical student tutors were at an advantage during the preparatory and teaching phase since from their peer tutoring experience they knew about the procedures and features of practical tutorials in medicine and found themselves in a familiar setting. The nursing student tutors had to adjust to the role of teacher, something that seems to have drawn on considerable psychological and cognitive resources (application of newly acquired teaching skills, strategies to strengthen self-esteem and authority as teacher, tolerance for ambiguity regarding tutee reactions). The definition of the tutor role appears to preempt group membership as nurse in differentiation to physician in certain areas. Above all in the assessment of professional superiority, pre-existing attitudes were accepted and applied in these cases, meaning that the medical students were perceived as having authority about tutorial content. In the context of the project the nursing student tutors were at a disadvantage in relation to the medical student tutors since they lacked prior teaching experience placing them in another power structure than the institutional hierarchy of the hospital setting with the associated differences in status [[21], S.48]. The phenomena connected with this during the project cannot be traced with certainty to the professional roles (in light of the situation here it is not even probable). The university students who were placed in the role of mentor due to the gap in the level of experience between the tutors were not required to activate positions of status related to professional identity to create inequality. It must be emphasized here that the university students are not accused of intending to create a discrepancy of status; however, this cannot be analyzed based on the particular conditions. The statements of the nursing student tutors indicate the opposite since the university student tutors were perceived as being supportive by the nursing students. It is also possible that any differences in age were significant in negotiating and assigning roles.

The following conclusion can be drawn from the results and observations:

- Both vocational and university student tutors should already have experience with peer teaching and be familiar with the teaching environment in order to concentrate fully on the special aspects of IPL.

Not only from a teaching standpoint is equality in this regard a necessity. In the interviews demands were made that the nursing content of each tutorial be defined and clearer ideas be given by the vocational schools about method and manner of presentation; this could then also be made accessible to the medical student tutors (interview 1). This leads to the second recommendation:

- Medical and nursing content should be standardized in the same manner for each tutorial. Each tutor should be familiar with the requirements expected of and material covered by the other professional group.

If these conditions are met, focus can be placed by both tutor groups on the particular aspects of interprofessional education and interprofessional collaboration. The design of the individual tutorials in the MITZ project took these aspects into account very differently. In addition to the joint performance of specific tasks, legal aspects concerning responsibility and the occasional communication issue were brought up during the collaboration; however, not always covered in the desired depth. In response to this, we make the following recommendation:

- Prior to preparing their tutorials, the tutors should be made aware of special aspects of interprofessional teamwork and the imparting of this information should also be defined. This applies mostly to legal and communication issues, but also to aspects of IPC governed by group dynamics or institutions, along with error management and feedback.

While group membership appears to play a role during preparation and teaching the tutorial only in terms of subject-specific aspects, stronger effects can be found on the social level. This is made clear in statements to the effect that there was only one tutorial-specific or inner-professional exchange took place between the tutors. The characteristic of being a tutor is not sufficient for generating a more comprehensive ingroup definition. For this reason, the following is recommended:

- The tutors should meet as often and for as long as possible as a large group and receive joint teacher or content-specific training. General rehearsals or events, such as semester parties, should also be held to help create a feeling a being a group. At MITZ attempts are being made to strengthen feelings of belonging together by wearing uniforms (green shirts).

This touches on the broadest conclusion of the project, not only in reference to the tutors but also the tutees:

- Contact is the basis for reflection on attitudes and stereotypes and the acquisition of knowledge about the other professional group: “It was simply interesting because when studying medicine all you see are doctors, people like you so to speak, and being able to get to know others just as they are fresh from school, that there are actually a lot of similarities with yourself” (interview 1).

The entire project demonstrated that mixed tutor teams are valuable if the goal is designing and conducting interprofessional tutorials because the idea of interprofessional learning and collaboration can be exemplified and transmitted more easily to students. There is hope that this has a positive influence on IPC since the tutors could later act as multiplicators. When transferring the results to interprofessional work setting it is important to differentiate between the effect of joint training on the tutors and the effects on the students attending the tutorials. If a pre-existing openness to interprofessional collaboration and awareness of stereotypes can be assumed in the selection of tutors, then this must be differentiated more clearly on the part of the tutees. Accordingly, the long-term effects of interprofessional tutorials on the tutees should be considered a further subject for research that cannot be addressed in this paper.

### Strengths and weakness of the study

The method applied in this study consciously aims to approach the topic of interprofessional tutor training in a manner not guided by research hypotheses and attempts to gain theoretical implications from the data. The limited generalizability that comes with small sample sizes was accepted in return for a deeper look at the cooperative processes between the tutors of both professional groups. The results can, however, be viewed as a reference point for further development and improvements to interprofessional tutorials and specifically tutor training. The extenuating circumstances during the project at MITZ appear to have had a significant influence on the behavior and perceptions of all of the tutors, as was explained in the previous section. For this reason a comparative study addressing the conclusions and recommendations discussed in this paper is desirable.

Furthermore, the socio-demographic profiles and (occupational) socialization of the interviewees were not taken into account by this study. Doing this could assist in better understanding some of the statements and underlying motives for behavior. It can be assumed that the scope and intensiveness of the practical experiences in dealing with the members of other professions varies greatly among the tutors. Due to previous training, prior education and different internships, making a general claim about the lack of contact between the two professions during the educational phase is only partially feasible as an explanation and would have to be differentiated by examining occupational socialization. The age difference is particularly large between medical students and the vocational students, a possible reason for different perceptions of various aspects and for differences in ability to reflect.

#### Outlook

This project will be carried out in the same form in the winter semester 2015/16. New university and vocational student tutors will be trained and will then modify and teach the regular tutorials. In addition to the development of tutor training, importance is placed on standardizing the educational content concerning interprofessional learning and collaboration.

In the future, interprofessional learning at the Faculty of Medicine Carl Gustav Carus at the Technische Universität Dresden, Germany, will play an increasingly greater role as an academic topic covered in practical courses. The focus is not only on integration of interprofessional courses into the required medical curriculum, but also in dentistry over the long term. As this is undertaken, the possibility exists for establishing testing formats to assess successful acquisition of competencies in interprofessional collaboration.

## Notes

^1^ Phenomena of intergroup relationships are caused by the process of social categorization occurring in the ingroup the person belongs to and outgroup that is compared with the ingroup in terms of at least one distinguishing difference [19].

## Competing interests

The authors declare that they have no competing interests.

## Figures and Tables

**Table 1:**
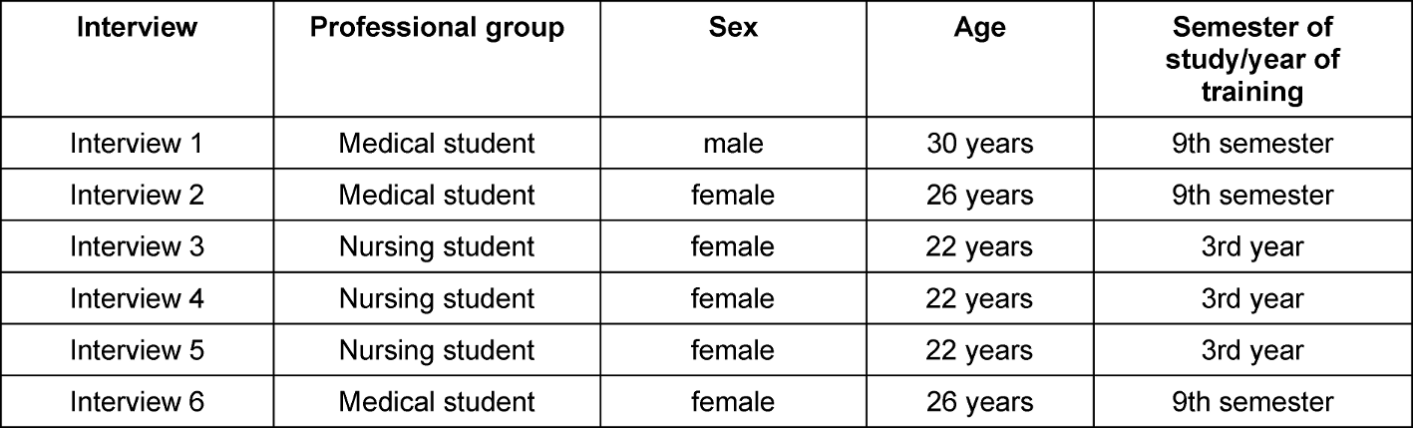
Characteristics of the interviewees

## References

[1] Mahler C, Gutman T, Karstens S, Joos S (2014). Begrifflichkeiten für die Zusammenarbeit in den Gesundheitsberufen: Definition und gängige Praxis. GMS Z Med Ausbild.

[2] Bartholomeyczik S, Donath E, Schmidt S, Rieger MA, Berger E, Wittich A, Dieterle WE (2008). Bericht "Arbeitsbedingungen im Krankenhaus".

[3] Knoll M, Lendner I (2008). "...dann wird er halt operiert und es ist keine Blutgruppe da!": Interprofessionelle Kommunikation von Pflegenden einer internistischen Intensivstation. Pflege.

[4] Antoni CH (2010). Interprofessionelle Teamarbeit im Gesundheitsbereich. Z Evid Fortbild Qual Gesundheitswes.

[5] Sieger M, Ertl-Schmuck R, Bögemann-Großheim E (2010). Interprofessionelles Lernen als Voraussetzung für interprofessionelles Handeln–am Beispiel eines interprofessionell angelegten Bildungs-und Entwicklungsprojektes für Gesundheitsberufe. Pflege Gesellschaft.

[6] Sachverständigenrat (2007). Gutachten 2007–Kooperation und Verantwortung. Voraussetzungen einer zielorientierten Gesundheitsversorgung. Dtsch Bundestag Drucksache.

[7] Walkenhorst U, Mahler C, Aistleithner R, Hahn EG, Kaap-Fröhlich S, Karstens S, Reiber K, Stock-Schröer B, Sottas B (2015). Positionspapier GMA-Ausschuss – Interprofessionelle Ausbildung in den Gesundheitsberufen". GMS Z Med Ausbild.

[8] Neitzke G (2005). Interprofessioneller Ethikunterricht. GMS Z Med Ausbild.

[9] Quandt M, Schmidt A, Segarra L, Beetz-Leipold C, Degirmenci Ü, Kornhuber J, Weih M (2010). Wahlfach Teamarbeit: Ergebnisse eines Pilotprojektes zur interprofessionellen und interdisziplinären Ausbildung mit formativem Team-OSCE (TOSCE).. GMS Z Med Ausbild.

[10] Kälble K (2013). Der Akademisierungsprozess der Pflege. Eine Zwischenbilanz im Kontext aktueller Entwicklungen und Herausforderungen. Bundesgesundheitsbl Gesundheitsforsch Gesundheitsschutz.

[11] Mahler C, Karstens S, Roos M, Szecsenyi J (2012). Interprofessionelle Ausbildung für eine patientenzentrierte Versorgung der Zukunft. Die Entwicklung eines Kompetenzprofils für den Bachelor-Studiengang "Interprofessionelle Gesundheitsversorgung". Z Evid Fortbild Qual Gesundheitswesen.

[12] Gardner S, George J, de Gibaja, M Gil, Jordan-Marsh M, Lind J, McCroskey J, Taylor H, Taylor-Dinwiddie S, Zlomik J (1998). A working paper on interprofessional education principles.

[13] Mey G, Mruck K, Mey G (2010). Interviews. Handbuch Qualitative Forschung in der Psychologie.

[14] Kuckartz U, Dresing T, Rädiker S, Stefen C (2008). Qualitative Evaluation: Der Einstieg in die Praxis.

[15] Nückles M, Bromme R (1998). Knowing what the others know: A study in interprofessional communication between nurses and medical doctors. Klin Pädiatrie.

[16] Ewers M (2012). Interprofessionalität als Schlüssel zum Erfolg. Public Health Forum.

[17] Hofmann I (2001). Schwierigkeiten im interprofessionellen Dialog zwischen ärztlichem und pflegerischem Kollegium. Pflege.

[18] Buxel H (2011). Was Pflegekräfte unzufrieden macht. Dtsch Ärztebl.

[19] Tajfel H, Billig MG, Bundy RP, Flament C (1971). Social categorization and intergroup behaviour. Eur J Soc Psychol.

[20] MacDonald MB, Bally JM, Ferguson LM, Murray BL, Fowler-Kerry SE, Anonson JM (2010). Knowledge of the professional role of others: A key interprofessional competency. Nurse Educ Pract.

[21] Wilkesmann, M (2008). Wissenstransfer im Krankenhaus. Institutionelle und strukturelle Voraussetzungen.

